# Diagnostic Performance of Serum Leucine-Rich Alpha-2-Glycoprotein 1 in Pediatric Acute Appendicitis: A Prospective Validation Study

**DOI:** 10.3390/biomedicines12081821

**Published:** 2024-08-11

**Authors:** Javier Arredondo Montero, Raquel Ros Briones, Amaya Fernández-Celis, Natalia López-Andrés, Nerea Martín-Calvo

**Affiliations:** 1 Pediatric Surgery Department, Complejo Asistencial Universitario de León, 24008 León, Spain; 2 Pediatric Surgery Department, Hospital Universitario de Navarra, 31008 Pamplona, Spain; 3 Cardiovascular Translational Research, NavarraBiomed (Miguel Servet Foundation), Hospital Universitario de Navarra, IdiSNA, Universidad Pública de Navarra (UPNA), 31008 Pamplona, Spain; 4 Department of Preventive Medicine and Public Health, School of Medicine, Universidad de Navarra, 31008 Pamplona, Spain; 5 IdiSNA, Instituto de Investigación Sanitaria de Navarra, 31008 Pamplona, Spain; 6 CIBER de Fisiopatología de la Obesidad y la Nutrición, Instituto de Salud Carlos III, 28029 Madrid, Spain

**Keywords:** LRG-1, serum, BIDIAP, prospective validation study, pediatric acute appendicitis, diagnostic performance, complicated appendicitis

## Abstract

**Introduction:** Leucine-rich alpha-2-glycoprotein 1(LRG-1) is a human protein that has shown potential usefulness as a biomarker for diagnosing pediatric acute appendicitis (PAA). This study aims to validate the diagnostic performance of serum LRG-1 in PAA. **Material and Methods:** This work is a subgroup analysis from BIDIAP (BIomarkers for DIagnosing Appendicitis in Pediatrics), a prospective single-center observational cohort, to validate serum LRG-1 as a diagnostic tool in PAA. This analysis included 200 patients, divided into three groups: (1) healthy patients undergoing major outpatient surgery (*n* = 56), (2) patients with non-surgical abdominal pain (*n* = 52), and (3) patients with a confirmed diagnosis of PAA (*n* = 92). Patients in group 3 were divided into complicated and uncomplicated PAA. In all patients, a serum sample was obtained during recruitment, and LRG-1 concentration was determined by Enzyme-Linked ImmunoSorbent Assay (ELISA). Comparative statistical analyses were performed using the Mann–Whitney U, Kruskal–Wallis, and Fisher’s exact tests. The area under the receiver operating characteristic curves (AUC) was calculated for all pertinent analyses. **Results:** Serum LRG-1 values, expressed as median (interquartile range) were 23,145 (18,246–27,453) ng/mL in group 1, 27,655 (21,151–38,795) ng/mL in group 2 and 40,409 (32,631–53,655) ng/mL in group 3 (*p* < 0.0001). Concerning the type of appendicitis, the serum LRG-1 values obtained were 38,686 (31,804–48,816) ng/mL in the uncomplicated PAA group and 51,857 (34,013–64,202) ng/mL in the complicated PAA group (*p* = 0.02). The area under the curve (AUC) obtained (group 2 vs. 3) was 0.75 (95% CI 0.67–0.84). For the discrimination between complicated and uncomplicated PAA, the AUC obtained was 0.66 (95% CI 0.52–0.79). **Conclusions:** This work establishes normative health ranges for serum LRG-1 values in the pediatric population and shows that serum LRG-1 could be a potentially helpful tool for diagnosing PAA in the future. Future prospective multicenter studies, with the parallel evaluation of urinary and salivary LRG-1, are necessary to assess the implementability of this molecule in actual clinical practice.

## 1. Introduction

Pediatric acute appendicitis (PAA) is the most frequent urgent abdominal surgical pathology in the world [1]. Although significant advances have been made in therapeutic terms at the expense of minimally invasive surgical techniques, there is still a considerable rate of misdiagnosis concerning this pathology, which in turn leads to substantial morbidity and mortality and an exponential increase in related healthcare costs [2]. At present, there is no single diagnostic test for this entity. Regarding diagnostic imaging tools, abdominal ultrasound (US) (including point-of-care ultrasound or POCUS) has demonstrated an excellent diagnostic yield in contemporary medical literature reports [3]. Similarly, low-radiation computed tomography (CT) has shown outstanding diagnostic performances in this context [4]. However, US is operator-dependent, requiring a specific training curve, and does not always allow for the identification of the cecal appendix; CT is a significant source of healthcare resource expenditure. Likewise, although CT radiation doses have been optimized and reduced during the last few years, it is still not recommended as a first-line test because the pediatric population is a particularly vulnerable group. Finally, magnetic resonance imaging (MRI) is a valuable tool in this pathology [5], but in many cases, patients require sedation for its utilization, and there is not always a radiology team available for its urgent application.

There are multiple validated multivariable scores for diagnosing PAA. Concerning the pediatric population, the Appendicitis Inflammatory Response (AIR) score and the Pediatric Appendicitis Risk Calculator (pARC) are the ones that have shown the best diagnostic performance in recent studies [6]. These tools stand out for their high areas under the curve (close to 0.90, depending on the study). The BIDIAP (BIomarkers for DIagnosing Appendicitis in Pediatrics) index, a recently published score demonstrating diagnostic performance and simplicity superior to precedents, is also relevant to this study. However, it has not yet been validated [7].

Many potential molecules have been studied and validated in recent years as valuable tools for diagnosing PAA and discriminating between complicated and uncomplicated PAA. On the one hand, hemogram ratios (such as the neutrophil-to-lymphocyte ratio, the platelet-to-lymphocyte ratio, and the systemic–immune-inflammatory index) stand out. These ratios are characterized by the ease and speed with which they can be obtained and their low cost. They have demonstrated high diagnostic yields and are a promising line of research in this pathology [7,8]. On the other hand, specific proinflammatory molecules such as Interleukin-6 have been extensively studied in this context, demonstrating usefulness for diagnosing PAA [9,10]. However, their implementation in clinical practice is often complex due to their economic cost and associated processing time.

Leucine-rich alpha-2-glycoprotein 1(LRG-1) is a human protein encoded by the LRG1 gene and is involved in protein-protein interaction, signal transduction, and cell adhesion and development. LRG-1 is expressed during granulocyte differentiation [11]. LRG1 is an acute phase reactant associated with multiple systemic inflammatory processes and acute bacterial infections [12]. Multiple proinflammatory cytokines, including Interleukin-6, induce LRG1 hepatic synthesis [12]. A recent systematic review and a meta-analysis showed significantly higher serum and urinary levels of LRG-1 in patients with PAA compared to controls. The potential usefulness of this biomarker for diagnosing PAA in saliva was also demonstrated in a pilot study [13]. It was subsequently confirmed in a prospective clinical study by Tintor et al. [14]. This study aims to validate the diagnostic performance of serum LRG-1 in pediatric acute appendicitis.

## 2. Material and Methods

### 2.1. Ethics

The Institutional Review Board of our center approved this project under code PI_2020/112. This research was conducted following the principles of the Declaration of Helsinki (2013 statement). All participants’ parents or legal representatives signed an informed consent form before the inclusion in the study.

### 2.2. Study Design

The present study is a subgroup analysis of the BIDIAP cohort [7]. BIDIAP was an observational prospective cohort designed to evaluate a panel of biomarkers as potential diagnostic tools in PAA.

The patients included in this study were divided into three groups: (1) patients with no underlying pathology who underwent scheduled outpatient surgery, such as circumcision or inguinal hernia surgery; (2) patients with acute abdominal pain who were attended to at the Emergency Department and in whom a diagnosis of acute appendicitis was finally excluded—also defined as non-surgical abdominal pain (NSAP), and (3) patients with a histologically confirmed diagnosis of acute appendicitis (PAA). For further analysis, patients in group 3 were stratified into non-complicated PAA (congestive, phlegmonous, or suppurative appendicitis) (from now on referred to as NCAA) and complicated PAA (gangrenous or perforated appendicitis) (from now on referred to as CAA) based on the histopathological classification of the appendix.

We defined “congestive appendicitis” as a polymorphonuclear infiltration of the appendix without invasion of the lamina propria. We defined “Phlegmonous or suppurative appendicitis” as a polymorphonuclear infiltration of the appendix with invasion of the lamina propria. We defined “Gangrenous appendicitis” as a polymorphonuclear infiltration of the appendix with invasion of the lamina propria and/or mural necrosis. Lastly, we defined “Perforated appendicitis” as any of the previous alterations with the presence of micro- or macroscopic perforation of the appendix [15]. This histological evaluation was performed in all cases by the same pathologist, blinded to the patient’s group.

Recruitment was carried out by convenience, when members of the research team were available. Strict inclusion and exclusion criteria were defined to minimize the risk of selection bias. In the center and period where the study was carried out, no non-operative management of PAA was performed. All patients with this diagnosis were treated surgically.

Inclusion criteria for this study were patients aged 0 to 14 years who presented to our pediatric Emergency Department (ED) with acute abdominal pain suggestive of acute appendicitis (pain initially in the mesogastric region and later radiating to the right lower quadrant or direct onset of pain in the right lower quadrant) of less than five days’ duration, associated with at least one of the following symptoms: hyporexia, nausea, vomiting, fever, diarrhea. The proposed age range corresponded to the pediatric care criteria established in our center, with patients 15 years of age and older being treated in the Emergency Department for adults.

Exclusion criteria for this study were: (1) clear suspicion of acute appendicitis or patients with clinical instability not requiring additional preoperative testing before surgery; (2) history of hematological disorders; (3) previously known metastatic malignancy; (4) active autoimmune diseases; (5) previous appendectomy; (6) immunosuppressive treatment within 28 days before enrollment; (7) systemic steroid treatment in the 14 days before enrollment; (8) abdominal trauma before enrollment.

All patients with NSAP were contacted two weeks after enrollment to confirm that they had not been diagnosed with PAA during this period. Patients were recruited when the investigators were available at the center. The recruitment period was from February to December 2021.

Sociodemographic and clinical information was collected upon recruitment during the patient’s stay in the ED. Analytical, surgical, radiological, and histological information was extracted from the participants’ clinical records.

### 2.3. Primary and Secondary Outcomes

The primary outcome of this subgroup analysis was to evaluate the diagnostic performance of serum LRG-1 in distinguishing between PAA and NSAP. The secondary outcomes were: (1) to evaluate the ability of serum LRG-1 to discriminate between complicated and uncomplicated PAA and (2) to establish a normative health rank for serum LRG-1 in the pediatric population.

### 2.4. Sample Size Calculation

Based on the preceding literature, and especially the work of Kharbanda et al. [16], we estimated that for a power of 0.8 and an alpha error of 0.05 (two-sided), a sample size of 49 patients per group would be required (PAA vs. NSAP).

### 2.5. Analyzed Variables

The age, sex, and weight of the patients were recorded in terms of the sociodemographic variables. Regarding the clinical variables, the hours since the onset of pain, the presence of fever at home, the number of diarrheal stools, the presence or absence of urinary symptoms, the number of emetic episodes, and the presence of hyporexia were included. Regarding the analytical variables, baseline serum LRG-1 values were collected in the three groups, and serum LRG-1 values were collected at 12 h post-surgery in the PAA group. The rest of the routine analytical variables (leukocytes, C-reactive protein, etc.) are available in previous publications of the BIDIAP cohort [7,17].

### 2.6. Sample Collection and Determination of LRG-1

Out of the 214 patients enrolled in the BIDIAP cohort, a venous blood sample with a vacutainer tube with separator gel (3.5 mL) was obtained, and serum LRG-1 was determined in 200. In the case of group 1 (healthy controls), samples were drawn during anesthetic induction and before the start of scheduled outpatient surgery. In the case of groups 2 (NSAP) and 3 (PAA), samples were drawn upon the arrival of the patients to the ED. Laboratory staff blinded to the patient group processed serum samples according to standard procedures. For the determination of serum LRG-1, an Invitrogen™ Human LRG-1 ELISA Kit from Thermo Fisher Scientific Inc. (Waltham, MA, USA) was used. The manufacturer’s instructions for ELISA analysis were followed. All determinations were obtained in ng/mL.

### 2.7. Statistical Analysis

Concerning descriptive statistics, we used median and interquartile range or mean and standard deviation for the quantitative variables and proportions for the categorical variables. The Kolmogórov–Smirnov test was used to assess the normality of the quantitative variables. Kruskal–Wallis, Mann–Whitney U and Fisher’s exact tests were used to compare sociodemographic and clinical variables between groups. To calculate serum LRG-1 capacity to distinguish between NASP and PAA and complicated and non-complicated PAA, we calculated the area under the receiver operating characteristic curves (ROC). The serum LRG-1 value with the highest Youden index (J) was chosen as the optimal cut-off. Alternative cut-offs were included, prioritizing sensitivity and specificity for illustrative purposes. Finally, a correlation analysis was carried out using Pearson and Spearman’s rank correlation coefficients. Correlation analyses were adjusted using Bonferroni correction where necessary.

A *p*-value < 0.05 (two tails) was considered statistically significant. All statistical analyses were carried out using STATA 17.0 (StataCorp LCC).

## 3. Results

### 3.1. Sociodemographic and Clinical Characteristics

This subgroup analysis included 200 patients divided into three groups: (1) patients with no underlying pathology who underwent scheduled outpatient surgery such as circumcision or inguinal hernia surgery (*n* = 56); (2) patients with NSAP (*n* = 52); and (3) patients with PAA (*n* = 92). Regarding the stratification of group 3, this analysis includes 64 patients with uncomplicated PAA and 28 patients with complicated PAA. 

Table 1 shows the sociodemographic characteristics of the patients by group. Statistically significant differences were found in the age, sex, and weight of the patients among the three groups. The age range was 3–14 years. Table 2 shows the clinical characteristics of the patients by group. Patients with PAA (group 3) presented a significantly higher number of emetic episodes compared to patients with NSAP (group 2). Also, patients with PAA presented significantly higher values of leukocytes, neutrophils, and C-reactive protein than those with NSAP. Table 3 shows the clinical characteristics of patients with PAA, whether NCAA or CAA. Patients with PAA presented a longer time of hours of pain from the onset of the condition until their ED assessment (*p* = 0.03), and a higher frequency of fever at home before their urgent assessment (*p* = 0.001). Also, patients with CAA presented significantly higher values of leukocytes, neutrophils, and C-reactive protein than those with NCAA. In relation to the final diagnoses of the NSAP group, the primary diagnoses identified were: “non-specific abdominal pain” “acute gastroenteritis”, “ileitis”, “mesenteric lymphadenitis”, “hemorrhagic follicular cyst”.

### 3.2. Serum LRG-1 Values

**Figure 1:**
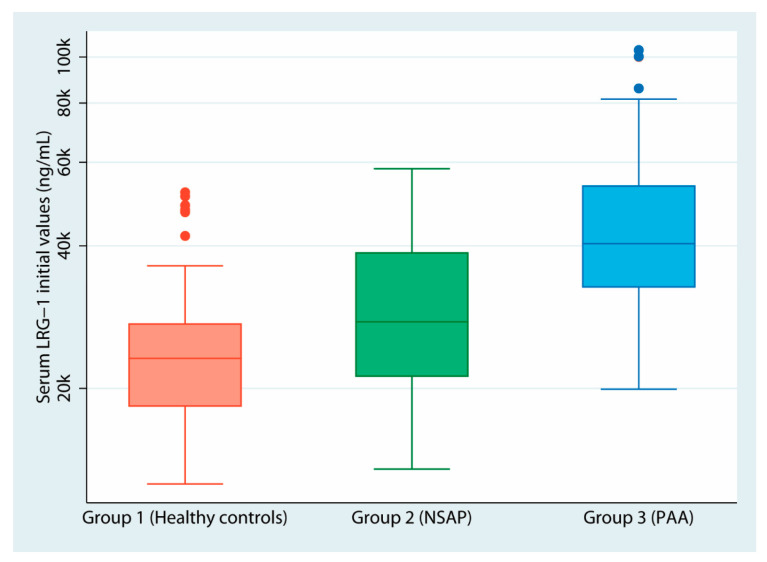
Box-plot representation of serum LRG-1 values per group. A logarithmic scale has been used to facilitate visualization, given the presence of outliers in groups 1 and 3. The designation “k” has been used after each integer to indicate that the values represented are multiples of 1000 (e.g., 20 k = 20,000).

Serum LRG-1 values by group are shown in Table 2 and Table 3. Statistically significant differences were found in baseline serum LRG-1 values between the three groups (*p* < 0.0001) and between the NSAP and PAA groups (*p* < 0.0001); Figure 1 depicts serum LRG-1 values by group using a logarithmic box plot. Concerning NCAA and CAA, statistically significant differences in baseline serum LRG-1 values were also found (*p* = 0.02). Similarly, LRG-1 was determined at 12 h post-surgery, and patients with CAA presented significantly higher values than those in the NCAA group (*p* = 0.002).

### 3.3. Diagnostic Performance of LRG-1 for Diagnosing Pediatric Acute Appendicitis 

Logistic regression analyses were performed to evaluate the diagnostic performance of serum LRG-1 comparing groups 1 (healthy controls) and 3 (PAA), groups 2 (NSAP) and 3 (PAA), and the NCAA and CAA groups. Excellent diagnostic performance was obtained when comparing healthy controls with patients with PAA, with an area under the curve (AUC) of 0.88 (95% CI 0.82–0.94). This AUC value indicates that serum LRG-1 strongly discriminates between healthy controls and PAA patients. When we compared the NSAP and PAA groups, a moderate–high diagnostic yield was obtained, with an AUC of 0.75 (95% CI 0.67–0.84), suggesting that serum LRG-1 can moderately discriminate between these two groups. Finally, a moderate diagnostic performance was obtained when comparing the NCAA and CAA groups, with an AUC of 0.66 (95% CI 0.52–0.79), indicating that serum LRG-1 can moderately discriminate between these two groups. When we compared the PAA and NSAP groups for leukocytes, neutrophils, and C-reactive protein, we obtained an AUC of 0.84 (95% CI 0.78–0.90), 0.86 (95% CI 0.80–0.92), and 0.74 (95% CI 0.65–0.83), respectively. When we compared the NCAA and CAA groups for leukocytes, neutrophils, and C-reactive protein, we obtained an AUC of 0.70 (95% CI 0.58–0.80), 0.70 (95% CI 0.59–0.81), and 0.71 (95% CI 0.59–0.84), respectively. 

When we analyzed Pearson and Spearman’s rank correlation coefficients, we obtained the following results: (1) correlation between leukocytes and serum LRG-1: Spearman’s Rho: 0.31 (*p* = 0.0002), Pearson’s: 0.29 (*p* = 0.0006); (2) correlation between neutrophils and serum LRG-1: Spearman’s Rho: 0.35 (*p* = <0.0001), Pearson’s: 0.31 (*p* = 0.0002); (3) correlation between CRP and serum LRG-1: Spearman’s Rho: 0.76c (*p* = <0.0001), Pearson’s: 0.57 (*p* = <0.0001). The scatter plots corresponding to the above analyses are shown in Supplementary Figure S1.

**Figure 2:**
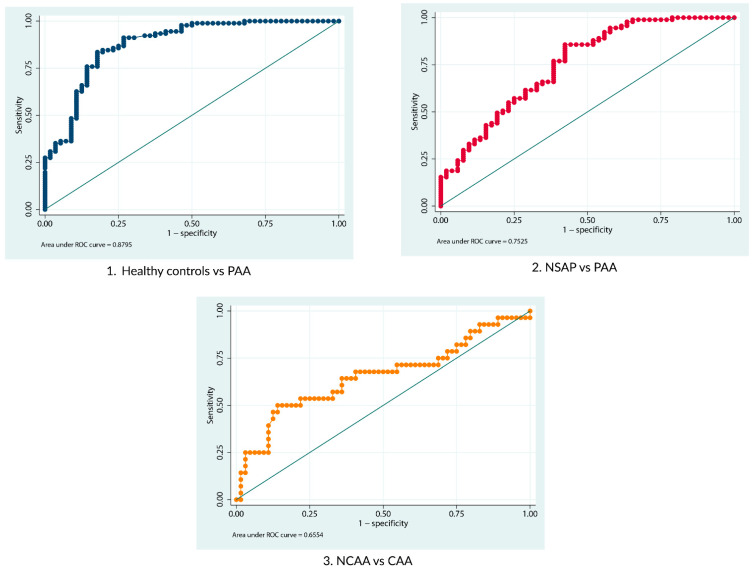
Graphical representation of the ROC curve for serum LRG-1. Above, Left: healthy controls vs. PAA; above, right: NSAP vs. PAA; bottom: NCAA vs. CAA.

Table 4 shows the analyses performed with their corresponding AUC, the proposed cut-offs, and their associated sensitivities and specificities. Figure 2 shows a graphical representation of the different ROC curves of each LRG-1 analysis.

## 4. Discussion

Identifying new diagnostic tools for PAA is a field of enormous scientific interest, given the high volume of patients suffering from this pathology and the severe consequences of its associated diagnostic errors. LRG-1, a molecule with potential as a diagnostic tool for PAA in pediatric populations, has shown promising results in previous evaluations [13,14,16,18,19,20,21,22,23,24]. In our study, we prospectively analyzed serum LRG-1 values in healthy children, patients with abdominal pain, and patients with confirmed PAA. Our results show statistically significant differences in serum LRG-1 values between the three groups and between patients with complicated and uncomplicated PAA. We also evaluated the diagnostic performance of this biomarker for discriminating between PAA and NSAP and for distinguishing between NCAA and CAA, obtaining a moderate–high performance (AUC 0.75) in the first case and a moderate performance (AUC 0.66) in the second case. Compared to the classical laboratory parameters routinely used for diagnosing PAA (leukocytes, neutrophils, and CRP), these still have a superior diagnostic performance. In this case, we believe that LRG-1, despite having a slightly lower diagnostic yield, still has the potential for diagnosing this entity, given its non-invasive character when determined in urine or saliva.

Regarding the values obtained and the comparison with the previous literature, our results are consistent with those of other earlier works, such as those of Kharbanda et al. [16] and Kakar et al. [22]. Both studies found statistically significant differences between the control and PAA groups and between the NCAA and CAA groups. It should be considered that there is an important heterogeneity when reporting the serum LRG-1 results, since some authors express them in ng/mL (such as our working group or Kharbanda et al.) while other authors express the values in μg/mL (such as Kakar et al.). Kharbanda et al. reported a 75th percentile value for the PAA group of 144,734 ng/mL that, in the case of complicated PAA, reached 202,579 ng/mL. These values are three times our 75th percentile for the same group of patients. This reinforces the hypothesis that many of these biomarkers frequently present extreme values or outliers and justifies studies with larger sample sizes that establish normative ranges to facilitate the interpretation of the values obtained for their eventual application in clinical practice.

The AUC reported by Kharbanda et al. and Kakar et al. were 0.69 (95% CI 0.60–0.79) and 0.95 (95% CI 0.91–0.99), respectively. In the case of Kharbanda et al., controls similar to our group 2 (NSAP) were used, and the values reported were consistent with ours. We believe both our AUC and that reported by Kharbanda et al. may be a realistic approximation of the diagnostic capability of this molecule in actual clinical practice. In the case of Kakar et al., the controls were recruited in the Emergency Department. Still, they consisted of patients without urinary, gastrointestinal, or respiratory inflammatory processes, which justifies the overestimated diagnostic yield reported by the authors.

Regarding cut-offs for the CG vs. AA comparison, Kharbanda et al. proposed 40,150 ng/mL (40.15 ug/mL) with a sensitivity of 100% and a specificity of 35%. On the other hand, Kakar et al. proposed 51,690 ng/mL (51.69 ug/mL) with an associated sensitivity and specificity of 93.8% and 91.1%, respectively. In our case, the proposed cut-offs were 28,614 ng/mL to distinguish NSAP (CG) from PAA and 44,382 ng/mL to discriminate NCAA from CAA. For the NSAP vs. PAA cut-off, our sensitivity and specificity were 85.7% and 57.7%, respectively, and for the NCAA vs. CAA cut-off, our sensitivity and specificity were 64.3% and 64.1%, respectively. Our cut-offs were chosen by globally prioritizing the best diagnostic performance using the Youden index, thus showing a more proportionate sensitivity/specificity ratio than that reported by Kharbanda et al., in which sensitivity was prioritized. Again, we believe that those reported by Kakar et al. are inconsistent with reality and are due to an unrepresentative choice of the control group for the analyses.

It is pertinent to highlight the presence of a group of healthy ambulatory patients in our study, given that one of the main problems in this type of research is the absence of reference values or ranges for the new biomarkers under investigation, such as LRG-1. The presence of previous studies in adults does not imply that the pediatric baseline values will be comparable.

About the correlation analyses performed, we found a weak–moderate correlation between serum LRG-1 and leukocytes, a weak–moderate correlation between serum LRG-1 and neutrophils, and a strong correlation between serum LRG-1 and CRP. Although the strong correlation found between CRP and LRG-1 constitutes an exciting finding, It should be noted the correlation models employed have limitations: (1) Regarding biological variables (serum biomarkers), many of these molecules (such as CRP, IL-6, Pentraxin-3, or LRG-1) rise at different rates during the natural course of PAA, and this time course is also variable between patients. Likewise, although many of these molecules have common signaling pathways (such as the pentraxin family—which includes CRP—or IL-6), it cannot be assumed that the elevation of these molecules is proportional and symmetrical (i.e., there may be patients with a higher elevation of CRP and a lower elevation of IL-6 or Pentraxin-3, for example). It should also be noted that many of these molecules present significant outliers in certain patients, making interpreting the findings challenging. (2) Regarding the statistical model chosen, Pearson’s correlation assumes a linear relationship between the two variables, meaning that a change in one variable will result in a proportional change in the other. However, biological relationships are not always linear, specifically when addressing serum biomarkers. Pearson’s correlation coefficient also requires that both variables are normally distributed. If this assumption is violated (such as in the present case), the correlation may not accurately reflect the strength of the relationship. Also, from a statistical point of view, outliers may bias the results of this specific correlation analysis. Lastly, although using both tests (Pearson and Spearman) helps to better characterize the potential correlation between these biomarkers compared to using a single test, we believe these analyses should be interpreted cautiously.

LRG-1 stands out, as previous recent reviews have shown [13], for its potential usefulness as a non-invasive biomarker in saliva and urine. Regarding LRG-1 in saliva, there is precedent work published by Yap et al. in 2020 [23]. In it, the authors prospectively evaluated the diagnostic performance of salivary LRG-1 with statistically significant differences between PAA and control groups and with an AUC of 0.77 (95% CI 0.60–0.93). They proposed a cut-off of 0.33 ng/ug and a sensitivity and specificity of 35.3% and 100%, respectively. In the BIDIAP cohort, a pilot test was performed to obtain salivary samples from patients to determine the LRG-1 value and compare it with serum values. However, this line of research was unsuccessful. First, because the collection of saliva is complex: (1) Nursing staff and the patient’s parents refused to perform it in young children because of the risk of suffocation (the sample is obtained by sucking an absorbent piece of cotton for a few seconds), (2) the amount of saliva obtained by this process differs significantly from one patient to another, being in many cases insufficient, and (3) the experience of laboratory staff in processing this biological material was limited. Nevertheless, most of these aspects are salvageable, and if validated, the findings reported by Yap et al. are of great interest.

Urinary LRG-1 has greater evidence in the context of PAA and is emerging as a promising biomarker. The AUC reported in the literature (CG vs. PAA) ranges from 0.59 to 0.97, with most studies reporting statistically significant differences between groups [13]. However, there are inherent limitations for this specific biological sample, such as the difficulty in obtaining a urine sample in patients with suspected PAA in the Emergency Department (often, these patients present a moderate degree of dehydration) and the question of whether or not a creatinine adjustment is necessary for this determination.

Finally, regarding serum LRG-1, the validation work of Tintor et al. [24] also supports the potential use of serum LRG-1 as a molecule for diagnosing PAA, along with our results and the previously published systematic review and meta-analysis [13].

There is also an extensive and growing body of evidence regarding the role of this molecule in diagnosing acute appendicitis in adults [25,26,27]. It should be considered, however, that although Rainer et al.‘s work evaluating modified whole blood LRG1 mRNA levels showed promising results [25], some papers show mixed results and lack the usefulness of LRG-1 for the diagnosis of acute appendicitis [26,27]. This highlights the need to conduct studies exclusively on pediatric populations since it cannot be assumed that the results of adult populations will be extrapolated to pediatric populations.

The present work has significant strengths, such as its prospective character, rigorous statistical analysis, and comparative and reasoned discussion of the findings with the preceding literature. However, it has limitations, such as its single-center character and the absence of saliva and urine samples. The disparity in sample size between groups, with a limited sample size of the CAA group, is the result of convenience recruitment and should also be considered a study limitation. Lastly, given the increased tendency of the non-surgical treatment of PAA with antibiotics, the lack of representation of this subgroup of patients in this study can be considered a limitation.

In conclusion, serum LRG-1 could be, in the future, a potentially helpful molecule for diagnosing PAA. This work establishes normative values in healthy children for LRG-1 and provides robust data compatible with the preceding literature regarding the diagnostic performance of this molecule in PAA. Future prospective multicentric studies with a larger sample size and with the three biological samples (blood, saliva, and urine) from the same patients are necessary to clarify the role of this molecule in PAA and its real clinical applicability.

## Figures and Tables

**Table 1:** Sociodemographic characteristics of the patients.

Sociodemographics	Group 1 (Healthy Controls) *n* = 56	Group 2 (NSAP) *n* = 52	Group 3 (PAA) *n* = 92	Total	*p*-Value
Age (years)	8.68 (3.26)	11.09 (2.48)	9.6 (3.01)	9.73 (3.08)	<0.001
Sex, Male/Total (percentage)	46/56 (82.1%)	24/52 (46.2%)	59/92 (64.1%)	129/200 (64.5%)	<0.001
Weight, kg	35.75 (18.38)	45.36 (15.40)	35.77 (12.09)	38.27 (15.47)	<0.001

Numbers are mean (standard deviation) or number (percentage). NSAP: non-surgical abdominal pain, PAA: pediatric acute appendicitis.

**Table 2:** Clinical and analytic characterization of the cohort.

Clinical Variables	Healthy Controls (*n* = 56)	NSAP (*n* = 52)	PAA (*n* = 92)	*p*-Value
Hours since the onset of pain	-	31.58 (23.13)	27.14 (19.42)	0.38
Fever at home >37.8 °C, Yes/no/missing	-	15/37	29/62/1	0.85
Number of diarrheal stools	-	0.4 (1.21)	0.68 (2.48)	0.59
Urinary symptoms, Yes/no/missing	-	8/44	21/70/1	0.39
Number of emetic episodes	-	0.56 (1.96)	2.48 (2.49)	<0.001
Hyporexia, Yes/no/missing	-	35/15/2	71/17/4	0.21
Leukocytes (×10^9^/L) *	-	9.6 (7.8–12.5)	16.1 (13–18.8)	0.0001 ***
Neutrophils (×10^9^/L) *	-	5.9 (4.1–8.4)	13.2 (9.5–16.2)	0.0001 ***
C-Reactive Protein (mg/L)	-	1.7 (1–22.2)	26.9 (6.4–63.3)	0.0001 ***
Initial LRG-1 (ng/mL) *	23,145 (18,246–27,453)	27,655 (21,151–38,795)	40,409 (32,631–53,655)	<0.0001 ** <0.0001 ***

PAA: Pediatric acute appendicitis, LRG-1: Leucine-rich alpha-2-glycoprotein 1. Numbers are mean (standard deviation) or number (percentage). * Median, interquartile range. ** Three-group comparison (Kruskal–Wallis test). *** NSAP compared with PAA (Mann–Whitney U test).

**Table 3:** Clinical characteristics of NCAA and CAA groups.

Clinical Variables	NCAA (*n* = 64)	CAA (*n* = 28)	*p*-Value
Hours since the onset of pain	24.63 (18.72)	32.77 (20.12)	0.03
Fever at home >37.8 °C, Yes/no/ missing	13/50/1	16/12	0.001
Number of diarrheal stools	0.75 (2.82)	0.54 (1.50)	0.78
Urinary symptoms, Yes/no/missing	14/49/1	7/21	0.79
Number of emetic episodes	2.24 (2.31)	3.04 (2.81)	0.23
Hyporexia, Yes/no/missing	48/13/3	23/4/1	0.56
Leukocytes (×10^9^/L) *	15.2 (12.1–17.6)	18 (15.4–21.8)	0.002
Neutrophils (×10^9^/L) *	12.1 (8.8–15.3)	15.3 (12.9–17.6)	0.001
C-Reactive Protein (mg/L)	18.2 (4.7–40.1)	63.3 (17.4–108)	0.0006
Initial serum LRG-1 (ng/mL) *	38,686 (31,804–48,816)	51,857 (34,013–64,202)	0.02
Serum LRG-1 at 12 h post-surgery (ng/mL) *, **	41,226 (33,904–50,918)	60,432 (44,809–69,370)	0.002

NCAA: Non-complicated pediatric acute appendicitis, CAA: Complicated pediatric acute appendicitis, *LRG-1:* Leucine-rich alpha-2-glycoprotein 1. Numbers are mean (standard deviation) or number (percentage). * Median, interquartile range. ** This analysis included 16 patients with CAA and 34 with NCAA.

**Table 4:** Diagnostic performance of serum LRG-1 in the different stratified analyses.

Group Comparison	AUC Value	95% CI	Proposed Serum LRG-1 Cut Off (ng/mL)	Sensitivity (%)	Specificity (%)
Healthy Controls vs. PAA	0.88	0.82–0.94	25,610	91.2	73.2
NSAP vs. PAA	0.75	0.67–0.84	28,614	85.7	57.7
NCAA vs. CAA	0.66	0.52–0.79	44,382	64.3	64.1

AUC: area under the curve, CI: confidence interval, NSAP: non-surgical abdominal pain, PAA: pediatric acute appendicitis, NCAA: non-complicated pediatric acute appendicitis, CAA: complicated pediatric acute appendicitis, LRG-1: Leucine-rich alpha-2-glycoprotein 1.

## Data Availability

All data in this study are available upon justified request to the author through correspondence.

## Supplementary Materials

The following supporting information can be downloaded at: https://www.mdpi.com/article/10.3390/biomedicines12081821/s1, Figure S1: Scatter-plots for Serum LRG-1 initial values and (a) Leukocytes (b) Neutrophils (c) C-Reactive protein
