# Supplementary figures and images for: Diagnostic Performance of Serum Leucine-Rich Alpha-2-Glycoprotein 1 in Pediatric Acute Appendicitis: A Prospective Validation Study
Source: Biomedicines. 2024 Aug 11;12(8):1821. doi: 10.3390/biomedicines12081821 (PMC11352011; doi:10.3390/biomedicines12081821)

(a)

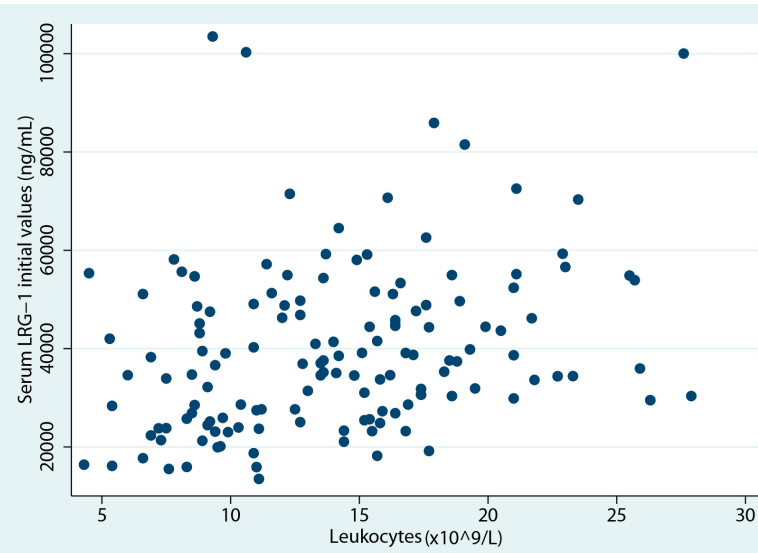

(b)

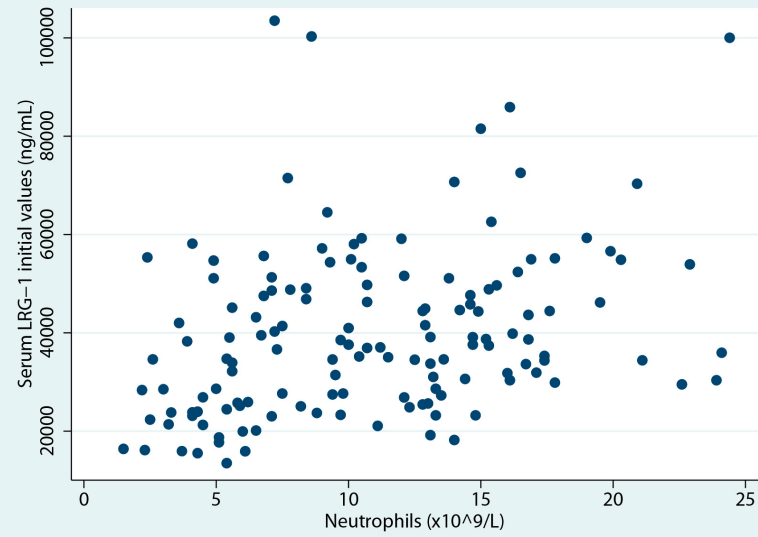

(c)

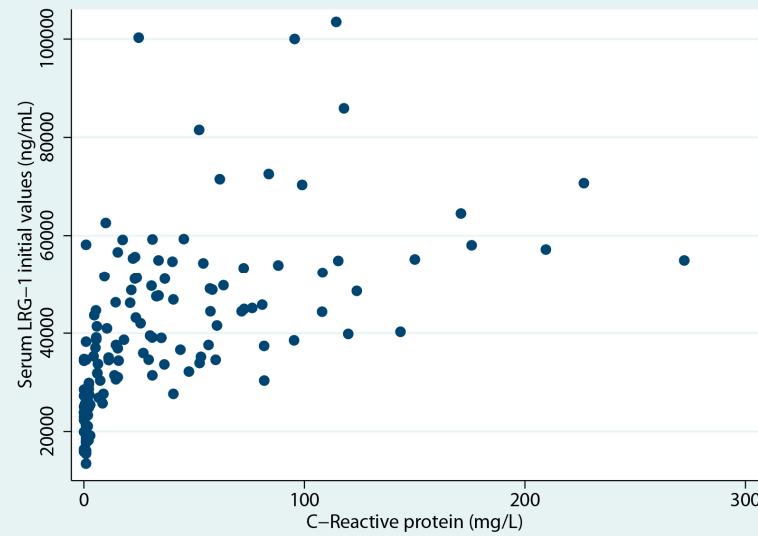

Supplement: Supplementary file 1 [file biomedicines-12-01821-s001.zip › biomedicines-3047343-supplementary.pdf]
